# Editorial: HIV-Induced Damage of B Cells and Production of HIV Neutralizing Antibodies

**DOI:** 10.3389/fimmu.2018.00297

**Published:** 2018-02-20

**Authors:** Francesca Chiodi, Gabriella Scarlatti

**Affiliations:** ^1^Department of Microbiology, Tumor and Cell Biology, Karolinska Institutet, Stockholm, Sweden; ^2^Viral Evolution and Transmission Unit, Division of Immunology, Transplantation and Infectious Diseases, San Raffaele Scientific Institute, Milan, Italy

**Keywords:** HIV, B cells, neutralizing antibodies, vaccination strategies, IgA

**Editorial on the Research Topic**

**HIV-Induced Damage of B Cells and Production of HIV Neutralizing Antibodies**

Memory B cells and long-lived plasma cells are pivotal for maintenance of serological memories to vaccines and infections. Studies in HIV-1-infected children and adults have shown that blood resting memory (RM) cells are reduced in number during HIV-1 infection and that their decline correlates with reduction of antibody (Ab) titers against childhood vaccinations [reviewed in Ref. (1, 2)]. Initiation of antiretroviral therapy (ART) shortly after infection restored RM cells to physiological levels in HIV-1-infected children and adults (3, 4) although very few studies have been conducted on this specific topic. One additional interesting feature of HIV-1 immunopathology is that exhausted memory B cells are expanded in circulation during HIV-1 infection; these exhausted cells comprise activated memory B (AM) and tissue-like memory (TLM) B cells, the latter displaying phenotypic similarities with tonsillar B cells (5, 6). The mechanism driving B cell abnormalities during HIV-1 infection remains poorly characterized. One possibility is that expression of inhibitory receptors on the surface of TLM B cells during HIV-1 infection, including the inhibitory receptor Fc receptor-like-4 (FCRL4), may engage specific pathway leading to inhibition of B cell proliferation and Ab production. Reverting *in vivo* the damage which HIV-1 exerts on B cells could possibly result in the production of adequate and persistent levels of HIV-1 neutralizing antibodies (NAbs) able to neutralize a broad range of HIV-1 isolates.

During the course of natural HIV-1 infection, humoral immune responses take place to HIV-1 epitopes resulting in specific Abs with non-neutralizing and neutralizing capacity. Only in a minority of infected individuals, Abs with the capacity to neutralize a broad range of HIV-1 isolates, called broad NAbs (bNAbs), can be detected after more than one year from primary infection. During the last decades a large number of potent HIV-1 bNAbs have been isolated from infected patients, which target the CD4 binding site, determinants within the V2 envelope (env) region, the V3 region or the gp120-gp41 interface region. These bNAbs have been tested in HIV-1 animal models, and phase I and II clinical studies have demonstrated safety in adults and children. Although some Fc-modifications are needed to increase the half-life of bNAbs, there is no doubt that they represent valuable tools in the contexts of HIV-1 prevention and treatment.

The clinical trials conducted with candidate HIV-1 vaccines targeting env showed that it is difficult to elicit high titers of HIV-1 bNAbs in humans. Accordingly, highly innovative approaches need to be applied to this field; integrated knowledge from vaccine design for other pathogens may accelerate the design of preventive or therapeutic HIV-1 vaccines with the property of inducing bNAbs.

In this research topic, we invited scientists to summarize the current state of knowledge on regulation and development of B cells and Abs responses during HIV-1 infection; 15 contributions were received comprising both reviews and original articles. A short introduction of these contributions follows.

Serological responses to vaccines and establishment of B cell memory is mediated through the interactions between Tfh cells and B cells in germinal centers (GCs). Pallikkuth et al. reviewed current knowledge on Tfh cells and B cells dysfunction in aging and HIV-1 infection. Chronic immune activation during HIV-1 infection affects the expression of molecules important for the function of Tfh cells and other T cell subsets including PD-1 and ICOS; T cell exhaustion could also take place as a result of protracted expression of inhibitory receptors. The number and function of circulating Tfh cells declined during HIV-1 infection but ART treatment resulted in increased frequencies of Tfh cells; however, upon these conditions the frequencies of RM cells remained low.

The expression of FcRL4 and IL-6 is increased in B cells during HIV-1 infection. Increased IL-6 expression leads to aberrant B cell differentiation and FcRL4 acts by dampening B cell receptor (BCR) signaling. Siewe et al. report that the expression of FcRL4 in viremic HIV-1-infected patients identifies an IL-6 producing pro-inflammatory B cell subset. In viremic patients AM and TLM cells expressed the highest levels of FcRL4 and IL-6; in addition, AM cells, followed by TLM cells, comprised the highest frequency of FcRL4^hi^IL-6^hi^ cells among B cell subpopulations. The authors present interesting mechanisms linking expression and signaling of FcLR4 with B cell damage and expression of inflammatory cytokines.

It is recommended that children born HIV-1-infected receive ART from birth and further studies should be conducted to analyze whether the damage to RM B cells is prevented by early ART introduction. Cotugno et al. reported that the frequencies of B cell subpopulations did not differ between controls and ART treated HIV-1-infected children who responded to treatment. Gene expression arrays performed on isolated B cells from selected HIV-1-infected patients revealed few differentially expressed genes in purified RM B cells when comparing controls and HIV-1-infected children. It is interesting however that 25 genes were differentially expressed in RM cells at baseline prior to influenza vaccination in the RM cells of vaccine non-responders as compared to vaccine responders. Gene profiles were also derived for AM cells in HIV-1-infected children and controls providing novel findings in the field of B cell damage.

B cells are involved in bone biology in health and disease. In her review, Titanji discusses the contribution of two cytokines produced by B cells, OPG, and RANKL, to HIV-1-induced bone loss. The members of the OPG/RANKL pathway are produced by a large number of cells present in several tissues of three major organ systems: skeletal, vascular, and immune systems. A strong link between joint destruction in rheumatoid arthritis (RA) and pathogenic RANKL producing B cells was found when patients with RA were treated with anti-CD20 Ab Rituximab. This treatment eliminated B cells and reduced also RANKL levels in synovium. Increased longevity in HIV-1-infected individuals receiving ART has been associated with higher prevalence of non-AIDS end-organ comorbidities including osteoporosis and cardiovascular diseases. During HIV-1 infection, the subset of TLM B cells, expanded as result of inflammation, has been linked to increased RANKL production. Both in HIV-1 transgenic rats and in untreated HIV-1-infected individuals an increased RANKL/OPG ratio was described, suggesting a link between the OPG/RANKL pathway and skeletal damage in HIV-1 infection. The exciting possibility of RANKL blockade by already available medicines during HIV-1 infection is discussed to reduce the impact of osteoporosis in aging patients.

Approximately 90%, of new HIV-1 acquisitions take place through mucosal contact. Kulkarni et al. describe how loss of B cells and plasma cells during HIV-1 infection results in a declined production of anti-HIV IgA responses at the mucosal sites. IgA present in mucosal secretions is produced at the mucosal site by plasma cells in the lamina propria and has a critical role for defense against pathogens. HIV-1 infection results in loss of integrity of mucosal barriers which are ultimately devoided of protective IgA and IgG; this scenario may contribute to superinfection with new HIV-1 strains and possibly give rise to the generation of new circulating recombinant HIV-1 forms. Passive immunization with either IgA or IgG is a potent tool to protect macaques from SIV infection at the mucosal level. When combining passive immunization of IgA and IgG, 100% protection was achieved although the mechanism of interactions between these two classes of Abs has yet not been elucidated. Vaccine strategies aimed at the induction of mucosal antibody responses needs to be further developed as preventive and therapeutic tool for HIV-1 infection.

Several HIV-1 bNAbs, especially the ones directed to the CD4 binding site and the gp120-gp41 interface region, also demonstrate specificity for self-antigens. Borhis et al. studied the interaction of B-cell-activating factor (BAFF) with its receptors BAFF-R and TACI. BAFF is a pivotal cytokine for B cell development, which, present at high levels during some autoimmune diseases, leads to increased rescue of self-reacting B cells. BAFF is also overproduced, in membrane-bound and soluble forms, during HIV-1 and SIV infections, where it may contribute to survival of immature transitional B cells, a population of cells which is enlarged during these infections. Based on these findings, the authors aim at understanding whether the interactions between BAFF and its receptors may be useful to enlarge pool of auto-reactive B cells producing bNAbs. These interesting findings point to the possibility that soluble TACI and BAFF-R may act as decoy receptors and that interactions between BAFF and its receptors may have a regulatory role in GC reaction acting on both B and Tfh cells.

Circulating biomarkers could be important to pin-point mechanisms which influence humoral immune responses and the development of HIV-1 bNAbs. Mabuka et al. examined whether dysfunctions taking place in B cell subpopulations during acute HIV-1 infection and the production of cytokines involved in B cell development (BAFF and CXCL13) can be linked to bNAbs development. Pathological changes in the composition of B cell subsets during acute HIV-1 infection were not predictive of the development of bNAbs. Interestingly, early high levels of CXCL13, but not BAFF, correlated with detectable bNAbs at 1-year postinfection. This finding calls for further studies to elucidate how elevated levels of the chemoattractant CXCL13, important for homing of Tfh and B cells to the GCs, may imprint the production of bNAbs.

Further intervention strategies, in addition to ART, may be needed to put an end to mother to child transmission (MTCT) of HIV-1. Douglas et al. reviewed the possibility that additional therapy opportunities for preventing HIV-1 MTCT may be provided by mapping the detailed specificity of protective maternal HIV-1 NAbs and characterizing the mechanisms through which maternal circulating viruses escape recognition from autologous NAbs. In the context of MTCT, vaccine strategies aimed at eliminating HIV-1 infection in children may only need to elicit Ab responses able to neutralize the virus pool from the mother to which the newborn is exposed. As shown in some of the reviewed studies, passively acquired ADCC mediating Abs from the HIV-1-infected mother may prolong survival in the infected infant; whether ADCC HIV-1 Abs need to be elicited by vaccines to protect children from HIV-1 MTCT should be further investigated.

Departing from the finding of naturally occurring Abs to the CC chemokine receptor 5 (CCR5) in healthy individuals and HIV-1-infected patients, Venuti et al. review the mechanism mediated by these Abs and suggest the use of anti-CCR5 Abs in therapeutic and vaccination strategies to combat viral infections. It is unclear why auto-Abs to CCR5 are produced in absence of autoimmune diseases, but a role for CCR5-Abs in homeostatic control is envisaged. Interestingly, CCR5 auto-Abs modulate CCR5 expression through a long-lasting internalization of this receptor and thus, may block HIV-1 transmission through CCR5, one of the two major chemokine receptors used by HIV-1 in attachment and penetration of target cells. Indeed, several novel immunization approaches have been used to induce anti-CCR5 Abs.

Soldemo et al. compared the induction of NAbs in chronically HIV-1-infected and immunized subjects. The HIV-1 bNAbs isolated from infected patients are generated through an extensive somatic hypermutation process as consequence of prolonged antigenic exposure upon chronic inflammation. Conventional immunization regimens of primates have so far failed to induce HIV-1 bNabs; the reasons for this failure is not known but the complex interplay between HIV-1 antigenic variability and B cell selection occurring *in vivo* may not be easy to mimic upon vaccination. Further studies in different animal models may define similarities and differences in germline antibody genes and expressed repertoires, thus paving the way to the design of effective HIV-1 vaccines.

The review by Molinos-Albert et al. focuses on the opportunities and challenges of utilizing the conserved membrane proximal external region (MPER) region within the Env gp41 protein to evoke bNAbs in HIV-1 immunization protocols. The MPER region, together with the gp41 fusion peptide, is involved in membrane destabilization. Structural and physical properties, including steric hindrance by gp120, do not render this region an easily accessible site to immunological responses. However, the isolation of some potent bNAbs against the MPER conserved region from HIV-1-infected subjects shows that, *in vivo*, this region can be a target of bNAbs. The authors present novel biochemical and immunological strategies on how to render the MPER site more accessible to B cell responses.

Non-neutralizing inhibitory Abs (nNAbs) may play an important role in decreasing HIV-1 load and may be useful in the context of HIV-1 protection. Mayr et al. present challenges and opportunities associated with HIV-1 nNAbs. These nNAbs can bind and capture infectious virus and form immune complexes and aggregates with the virus. Their inhibitory function is mediated through the binding of its Fc-domain to specific FcRs present at the surface of immune cells. Polymorphism of FcRs may pose a limitation to the development of HIV-1 vaccines aimed at inducing nNAbs. An interesting picture is emerging depicting the role that Fc-mediated phagocytosis of immune complexes may have in inducing immune activation and promoting adaptive antiviral responses.

In the review by Hua et al. the authors present the different scenarios where bNAbs may be of clinical utility ranging from preventing viral infection, enhancing therapeutic potential in acute infection and chronic infection. The pharmacological modalities of bNAbs action are multiple and vary from the capacity to enhance adaptive immune responses to potential reduction of virus reservoirs. There are however limitations to be dealt with before bNAbs can be introduced in clinical HIV-1 contexts; for example, selection of resistant viral populations, development of Ab responses directed to the administered bNAbs and risk of eliminating HIV-1 reservoirs in regeneration limited compartments. In this review engineering and biological approaches are widely discussed to overcome limitations to the use of bNAbs.

Modification of the structure of the immunogen is a front-line research topic to increase its capacity to induce and stimulate bNAb responses. Soldemo et al. present in their article how cross-linking of HIV-1 env trimers with glutaraldehyde (GLA) affects thermo-stability and exposure of nNAbs epitopes *in vitro* and env-specific IgG Ab responses *in vivo*. GLA fixation improved the stability of the env-trimers, however at the expense of a lower Ab response to the trimers upon repeated immunizations. Mice inoculated with GLA fixed trimers displayed a more Th2-skewed subclass profile as compared to animals inoculated with native trimers. Coadministration of adjuvants known to balance Th1/Th2 responses were not able to redirect this Th2-skewed profile.

Forsell et al. investigated a mechanism for epitope-specific regulation and maturation of B cell responses. The experimental set-up aimed at pin-pointing the profiles of GC B cell responses evoked by one injection with an env protein in a murine system and at understanding if injection with env-Abs could exert regulation of GC B cell responses in an epitope-specific manner. The results suggest that env-specific B cell responses are negatively regulated through epitope masking by high affinity Abs. Ab-mediated feedback to GC B cells may be effective only when GC B cells share the same specificity with an injected or circulating Ab. This proposed mechanism of Ab-mediated feedback, in addition to unraveling basic aspects of regulation of GC B cell responses, will be important in efforts aimed at developing effective HIV-1 vaccine.

It is our hope that the collection of articles presented in this research topic may be useful for new and experienced scholars in the field and add a piece to the complex puzzle of knowledge needed for the development of an HIV-1 vaccine.

## Author Contributions

FC and GS are responsible for the research topic: HIV-induced damage of B Cells and Production of HIV Neutralizing Antibodies (5357).

## Conflict of Interest Statement

The authors declare that the research was conducted in the absence of any commercial or financial relationships that could be construed as a potential conflict of interest.
